# Research on Adaptive Cooperative Positioning Algorithm for Underwater Robots Based on Dolphin Group Cooperative Mechanism

**DOI:** 10.3390/biomimetics11010082

**Published:** 2026-01-20

**Authors:** Shiwei Fan, Jiachong Chang, Zicheng Wang, Mingfeng Ding, Hongchao Sun, Yubo Zhao

**Affiliations:** 1 College of Computer and Control Engineering, Northeast Forestry University, Harbin 150040, China; fanshiwei@nefu.edu.cn (S.F.);; 2 College of Electronic Science, National University of Defense Technology, Changsha 410000, China; jiachong.chang@connect.polyu.hk; 3 Shenyang Military Representative Bureau of Naval Equipment Department, Harbin 150000, China; 4 College of Urban Rail Transit, Jilin Tiedao University, Jilin 132000, China

**Keywords:** factor graph, adaptive cooperative positioning, unmanned underwater vehicle

## Abstract

Inspired by the remarkable collaborative echolocation mechanisms of dolphin pods, the paper addresses the challenge of achieving high-precision cooperative positioning for clusters of unmanned underwater vehicles (UUVs) in complex marine environments. Cooperative positioning systems for UUVs typically rely on acoustic ranging information to correct positional errors. However, the propagation characteristics of underwater acoustic signals are susceptible to environmental disturbances, often resulting in non-Gaussian, heavy-tailed distributions of ranging noise. Additionally, the strong nonlinearity of the system and the limited observability of measurement information further constrain positioning accuracy. To tackle these issues, this paper innovatively proposes a Factor Graph-based Adaptive Cooperative Positioning Algorithm (FGAWSP) suitable for heavy-tailed noise environments. The method begins by constructing a factor graph model for UUV cooperative positioning to intuitively represent the probabilistic dependencies between system states and observed variables. Subsequently, a novel factor graph estimation mechanism integrating adaptive weights with the product algorithm is designed. By conducting online assessment of residual information, this mechanism dynamically adjusts the fusion weights of different measurements, thereby achieving robust handling of anomalous range values. Experimental results demonstrate that the proposed method reduces positioning errors by 22.31% compared to the traditional algorithm, validating the effectiveness of our approach.

## 1. Introduction

Biomimetics, the discipline of emulating nature’s models and systems to solve complex human challenges, has catalyzed breakthroughs across diverse engineering fields, from materials science to robotics [1,2,3,4]. In the realm of underwater navigation and acoustic sensing, marine mammals, particularly dolphins, have evolved sophisticated biological systems that far surpass the capabilities of many artificial technologies [5,6,7]. The ability of dolphins to work collaboratively brings new inspiration for cooperative navigation technology involving multiple unmanned underwater vehicles (UUVs).

Achieving high-precision positioning in complex underwater environments is fundamental for multi-UUV (unmanned underwater vehicle) collaborative systems to fulfill their missions [8,9]. For instance, in ocean depth measurement applications, accurate positioning ensures seamless coverage of target areas during survey tasks [10]. However, since Global Navigation Satellite Systems (GNSSs) cannot function properly underwater, high-precision positioning poses significant challenges. Acoustic signals, as the primary medium for underwater ranging and communication [11], have led many researchers to explore cooperative positioning schemes for multiple UUVs using long baselines [12], short baselines [13], and ultra-short baselines [14]. Yet, in practical applications, baseline deployment often proves difficult, resulting in notable limitations for acoustic positioning systems based on baselines. Inertial navigation systems, with their strong concealment and high autonomy, have been widely adopted in UUV positioning. Their high-precision positioning error can be controlled within 0.1% of the navigation distance [15], but they remain costly and challenging to scale up for large-scale deployment.

In recent years, significant progress has been made in navigation technology for UUVs. For example, Sagar et al.’s [16] review paper “State of the Art Navigation Systems and Sensors for Unmanned Underwater Vehicles” systematically reviews the latest developments in UUV navigation systems and sensors, covering various technologies from inertial navigation to acoustic sensors, providing a comprehensive reference framework for research in this field. However, as a review work, it focuses on describing the current situation and lacks in-depth analysis and verification of innovative methods. On the other hand, Zhang et al. [17] proposed a distributed fusion positioning method based on information geometry in “UUV Cluster Distributed Navigation Fusion Positioning Method with Information Geometry”, which achieved high-precision positioning of UUV clusters under free aggregation and dispersion through factor graph theory. Its robustness in dynamic marine environments still needs further verification. These two papers have, respectively, promoted research on UUV navigation from the perspectives of macro-level technology review and micro-level method innovation, providing a theoretical basis for this work while highlighting the necessity of developing light-weight and adaptive navigation solutions.

**Figure 1:**
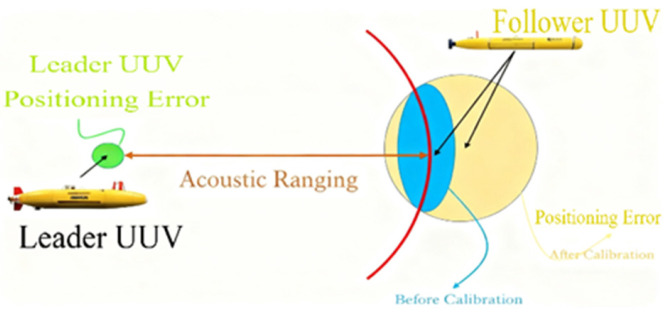
Schematic diagram of cooperative positioning system.

In recent years, the academic community has conducted extensive research on cooperative positioning technology for acoustic multi-autonomous underwater vehicles (UUVs), and the principle of cooperative positioning is shown in Figure 1. With the emergence of various cooperative positioning algorithms, classical Kalman filter (KF) methods have become inadequate due to the nonlinear characteristics of the system. Wang and Costanzi attempted to apply the EKF algorithm to the field of cooperative positioning [18,19]. However, EKF exhibits significant limitations, including linearization errors and Gaussian assumptions. Although studies show that the unscented Kalman filter (UKF) outperforms EKF in positioning accuracy, its excessive computational load restricts its application in real-time positioning [20].

To develop cooperative positioning algorithms with reduced estimation errors and computational overhead, graph optimization theory has garnered significant attention in recent years [21,22]. Factor graphs, as probabilistic graph models, visually represent the decomposition structure of global multivariable functions—functions composed of multiple local factors [23]. This graphical approach effectively reduces computational complexity and nonlinear errors in systems [24]. Compared to extended Kalman filter (EKF) and untracked Kalman filter (UKF), factor graphs enhance temporal correlation in measurement data by integrating all historical information through factor connections.

The statistical characteristics of acoustic ranging errors in UUVs are profoundly shaped by the complex underwater environment. Phenomena such as multipath propagation and sound speed profile variations can collectively distort the error distribution, leading to distinctly non-Gaussian, heavy-tailed characteristics and frequent outliers. Conventional Gaussian-based filters, including the extended Kalman filter (EKF) and unscented Kalman filter (UKF), which assume symmetric and light-tailed noise, are inherently mismatched to these real-world conditions. This mismatch often results in substantial estimation bias and, in the presence of significant outliers, can lead to filter divergence. While variational Bayesian filters [25] offer some robustness to uncertainty, they may still struggle to fully capture the pronounced heavy-tailed nature of the errors induced by the challenging underwater channel [26,27].

Interestingly, dolphin groups inherently exhibit remarkable resilience to similar challenges. When navigating or hunting collectively, they must discern their own echoes amidst the cacophony of pod members’ clicks and the ambient noise, effectively solving a “cocktail party problem” in real time. They achieve this through dynamic adjustments to their sonar parameters and advanced neural processing, demonstrating an innate capability for outlier rejection and adaptive signal processing that current algorithms strive to achieve.

The core biomimetic inspiration for this work stems from the sound wave beamforming mechanism observed in dolphins. Specifically, we focus on how dolphins overcome multipath effects, environmental noise, and other interferences by actively adjusting their acoustic signal characteristics and cooperative strategies among individuals, achieving robust communication and relative positioning. This dynamic and adaptive signal processing and collaborative strategy is the inspiration for the biomimetics design of the algorithm proposed in this article.

Inspired by the adaptive and collaborative nature of dolphin group echolocation, this paper proposes a Factor Graph-based Adaptive Cooperative Positioning Algorithm (FGAWSP) specifically designed for harsh underwater environments. The main contributions are threefold:

(1) Bio-inspired Robustness Mechanism: We introduce the concept of continuous, residual-based fault diagnosis into the factor graph framework, mirroring the adaptive signal processing observed in dolphin groups to achieve resilience against anomalous acoustic ranging information.

(2) Seamless Adaptive Weighting: Moving beyond binary acceptance or rejection of measurements, we design a novel weighting scheme that dynamically classifies ranging data into multiple trust levels (e.g., “trustworthy,” “partially trusted,” and “untrustworthy”), maximizing the use of valid information under heavy-tailed noise, much like a dolphin pod efficiently utilizes distributed acoustic information.

(3) Comprehensive Validation: The superior performance of the proposed FGAWSP algorithm is rigorously validated through numerical simulations and real-world experiments, demonstrating its potential to advance robust, biomimetic cooperative navigation systems.

The remainder of this paper is organized as follows: Section 2 details the problem formulation and the limitations of traditional methods. Section 3 elaborates on the proposed FGAWSP algorithm. Section 4 presents and discusses the experimental results. Finally, Section 5 concludes the paper.

## 2. Problem Statement

### 2.1. Construction of the System Model

The navigation and cooperative positioning of UUVs depend on a suite of onboard sensors. Leader UUVs are equipped with a high-precision Inertial Navigation System (INS) integrated with a Doppler Velocity Log (DVL) to estimate their position, velocity, and attitude. In contrast, follower UUVs typically utilize a DVL and a magnetic compass for this purpose. Furthermore, a depth sounder (or pressure sensor) on each vehicle provides precise depth measurements. The relative distance between UUVs, which is essential for cooperative positioning, is measured by acoustic modems or rangefinders. Given the accurate depth information, it is feasible to project the positional data and distance observations onto a horizontal plane, thereby reducing the cooperative positioning problem to a two-dimensional analysis. Since the acoustic rangefinders operate in three-dimensional space, a transformation matrix must be applied to project these measurements onto the horizontal plane of the navigation coordinate system. The specific transformation process is described by the following formula:

(1) $$V_{E}=C_{S}^{E}V_{S}$$

where *E* represents the geographic coordinate system, and *S* represents the carrier coordinate system. $V_{E}$ represents the three-dimensional velocity in the geographic coordinate system, $C_{S}^{E}$ represents the transformation matrix between the carrier coordinate system and the geographic coordinate system, $V_{S}$ represents the three-dimensional velocity in the carrier coordinate system.

Other three-dimensional physical quantities (such as the distance between the leader UUV and follower UUV) can also be converted to the geographic coordinate system in the same way. Subsequent research on cooperative positioning is conducted in the horizontal plane of the geographical coordinate system, thus transforming the three-dimensional problem into a two-dimensional problem. Therefore, in the kinematic model of an AUV in cooperative positioning, there is no need to consider the two horizontal attitude angles, resulting in a nonlinear motion equation as shown in Equation (2):

(2) $$\left\{\begin{array}{l} \dot{x}=v\cos\theta +w\sin\theta \\ \dot{y}=v\sin\theta -w\cos\theta \end{array}\right.$$

where (*x*, *y*) represents the coordinate of the UUV in the planar rectangular coordinate system, v represents the longitudinal velocity of the UUV in the carrier coordinate system, *w* represents the lateral velocity of the UUV in the carrier coordinate system, and θ represents the yaw angle of the UUV in the geographic coordinate system.

The coordinates of the UUV at time *k* are denoted as **x**_k_ = [*x*_k_, *y*_k_]^T^. At the same time, the UUV can measure the speed *v*_k_ at this moment using the odometer (the lateral speed is ignored in this study unless otherwise specified), and the yaw angle $\theta_{k}$ of the UUV at time *k* is measured using a compass. The sampling time between adjacent moments is Δ*t*. Based on Equation (2), the discrete motion model shown in Equation (3) can be obtained.

(3) $$\left\{\begin{array}{l} x_{k}^{-}=x_{k-1}+{\hat{v}}_{k}\Delta t\cos\theta_{k}\\ y_{k}^{-}=y_{k-1}+{\hat{v}}_{k}\Delta t\sin\theta_{k} \end{array}\right.$$

The paper focuses on a leader–follower UUV cooperative positioning system. The leader UUV is equipped with high-precision navigation equipment, while the follower UUV (equipped with low-precision navigation devices) receives position and observation data from the leader during cooperative positioning. Through real-time communication between UUVs, the follower UUV acquires both positional and observational data from the leader UUV. Since the observation data in the underwater cooperative positioning system are based on relative distance, the observation equation for the system can be expressed as Equation (4):

(4) $$z_{k}^{3}=\sqrt{{\left(x_{k}-x_{k}^{m}\right)}^{2}+{\left(y_{k}-y_{k}^{m}\right)}^{2}+{\left(h_{k}-h_{k}^{m}\right)}^{2}}$$

where $\left(x_{k}^{m},y_{k}^{m}\right)$ represents the coordinate of the leader UUV at time *k*, $z_{k}^{3}$ represents the three-dimensional distance information between the leader UUV and follower UUV measured by the acoustic modem at time *k*, $h_{k}$ represents the depth information of the UUV at time *k*, and $h_{k}^{m}$ indicates the depth information of the leader UUV at time *k*.

Since the depth of the UUV can be accurately measured by a bathymetric device, and the computational load in two-dimensional space is significantly lower than that in three-dimensional space, it is only necessary to calculate the two-dimensional position of the UUV on the horizontal plane. The depth sensors carried by UUVs can provide depth measurement values far higher than that of horizontal positioning. As shown in Equation (5), the process of projecting a three-dimensional slant distance onto a two-dimensional horizontal plane distance strongly relies on accurate depth difference measurements. In the current model, we assume that depth errors can be ignored, which is true in the application scenarios of high-precision depth sensors commonly used in hydrological measurement tasks (errors can usually be controlled at the decimeter or even centimeter level). Under these conditions, reducing the dimensionality of a three-dimensional problem to two-dimensional processing can significantly reduce computational complexity and avoid filter convergence issues caused by the expansion of three-dimensional spatial state dimensions. Therefore, the observed information obtained at this moment also needs to be converted into distance information in two-dimensional space, as shown in Equation (5):

(5) $$z_{k}=\sqrt{{\left(z_{k}^{3}\right)}^{2}-{\left(h_{k}-h_{k}^{m}\right)}^{2}}$$

where $z_{k}$ represents the distance information between the leader UUV and follower UUV in the two-dimensional plane after conversion at time *k*.

With the increase in autonomous positioning time for follower UUV, the error in yaw calculation grows significantly. To minimize positioning errors within a tolerable range, the follower UUV must obtain position information and observation information from the leader UUV. During actual operations, due to the complex underwater acoustic propagation environment, as well as instrument errors arising from the inherent physical properties and manufacturing constraints of the equipment, the measured values are adversely affected. In such cases, Equation (6) can be applied to quantify these discrepancies:

(6) $$z_{k}=\sqrt{{\left(x_{k}-x_{k}^{m}\right)}^{2}+{\left(y_{k}-y_{k}^{m}\right)}^{2}}+\lambda_{k}$$

where $\lambda_{k}$ represents the observation error of the distance measurement at time *k*. It is assumed to be a Gaussian distribution, with a mean of 0 and a variance of $\sigma_{z}^{2}$.

### 2.2. Traditional Chi-Square Test Principle

In traditional chi-square testing, statistical quantities can be constructed from the new measurement and its mean square variance array

(7) $$\lambda_{k}=r_{k}^{T}A_{k}^{-1}r_{k}$$

where $A_{k}$ represent the measurement prediction error (i.e., innovation) and its mean square error matrix, respectively. Theoretically, the innovation sequence $r_{k}$ is zero-mean white noise and $\lambda_{k}$ follows a chi-squared distribution with *m* degrees of freedom, that is $\lambda_{k}\sim \chi^{2}(m)$. In the case of normal measurements, the value of the statistic $\lambda_{k}$ should be relatively small; whereas if there is an anomaly in the measurements (for example, ranging information is affected by multipath effects), $\lambda_{k}$ will become larger. The determination of whether the measurements are normal or not is generally based on a selected threshold $Y_{Dm}$ as the judgment limit

(8) λk⩽YDmλk>YDm ρk=1ρk=0

This is the measurement fault chi-square test principle of the Kalman filter. During the filtering process, statistical quantities can be calculated to monitor measurement abnormalities in real time based on their magnitude, thereby determining whether to update measurements. The measurement and its mean square variance matrix update equations can be rewritten as follows

(9) $$\left\{\begin{array}{l} {\hat{X}}_{k}={\hat{X}}_{k/k-1}+\sqrt{\rho_{k}}K_{k}r_{k}\\ P_{k}=\left(I-\rho_{k}K_{k}H_{k}\right)P_{k/k-1} \end{array}\right.$$

where ${\hat{X}}_{k}$ represents the state variables of the system at time *k*. $P_{k}$ represents the covariance matrix corresponding to the state variables at time *k*, $K_{k}$ represents the filtering gain at time *k*, I represents the unit matrix, and $H_{k}$ represents the system measurement matrix at time *k*. Obviously, when $\rho_{k}=1$, that is $\lambda_{k}⩽Y_{Dm}$, normal Kalman filter measurement update is performed. When $\rho_{k}=0$, the measurement update is abandoned, and the valid information in the measurement data is discarded, achieving the effect of isolating abnormal measurements.

## 3. Cooperative Positioning Algorithm with Adaptive Estimation

For a UUV cooperative positioning system, data fusion is essential when coordinating positioning between vessels. This process requires integrating the primary vessel’s position information with distance data between leader and follower UUVs. When employing factor graphs and the sum-product algorithm for position estimation, it becomes necessary to utilize the probability distributions of various data sources. To achieve this, constructing a probabilistic density function for underwater acoustic ranging serves as crucial reliability information within the sum-product algorithm framework. Consequently, the accuracy of position estimation ultimately depends on how effectively and comprehensively these available reliability parameters are utilized.

While previous work has reduced three-dimensional cooperative positioning to a two-dimensional problem, this study employs factor graphs to further simplify it into a one-dimensional framework [28,29]. The problem is categorized into two distinct one-dimensional scenarios based on the geometric relationship between the lead vessel and follower vessels. These two one-dimensional problems are, respectively, represented by the x-coordinate group in the factor graph and the two primary node groups within the y-coordinate system.

Factor graphs are inherently non-directional. By definition, all neighboring nodes of a node have the opposite type. Most factor graph-based problems are solved through iterative transmission of “credibility information” between variable nodes and function nodes, implemented via the sum-product algorithm. This credibility information describes variables’ expected values, variances, and other relevant metrics. During iterative computation, the following principles govern the transmission of credibility information:

(1) The information sent from variable node x to a function node is the product of all information received at *x* except that from the function node.

(2) The information passed from a function node to a variable node is the product of all the information passed to the function node by the neighboring variable nodes other than the variable node and the function. Then, the integral of all relevant variables is obtained. For a discrete system, it can be expressed in the form of summation.

The above two criteria can be expressed as follows [30]:

(10) $$\begin{array}{l} \mu_{X_{k}\to \gamma_{j}}(x)=\prod_{h\in N_{X_{k}}\backslash \left\{\gamma_{j}\right\}}\mu_{h\to X_{k}(x)}\\ \mu_{\gamma_{j}\to X_{k}}(x)=\sum_{y\in \chi_{\tilde{V}\backslash \{k\}}}\gamma_{j}(y,x)\prod_{Y\in N_{\gamma_{j}\backslash \left\{X_{k}\right\}}}\mu_{Y\to \gamma_{j}\left(y_{j}\right)} \end{array}$$

where *X_k_* and *Y* represent variable nodes, $\gamma_{j}$ represents a function node, *x* and *y* denote the variable, $N_{X_{k}}$ denotes the set of function nodes connected to the variable node *X_k_*, $N_{\gamma_{j}}$ denotes the set of variable nodes connected to the function node $\gamma_{j}$, and $y_{j}$ represents the *j*th variable node connected to $\gamma_{j}$ (excluding *X_k_*).

According to the above two criteria, information can be passed through the factor graph, and the estimated value of a variable can be obtained via the product operation. The estimated value of the variable *x* can be expressed as follows:

(11) $$\mu (x)=\prod_{h\in N_{X_{k}}}\mu_{h\to X_{k}(x)}$$

The above criteria are the calculation process of the factor graph and product algorithm. Through the calculation of variables in the factor graph via the product algorithm, the estimated solution of the global problem can be obtained, which greatly reduces the algorithm complexity and improves computational efficiency.

**Figure 2:**
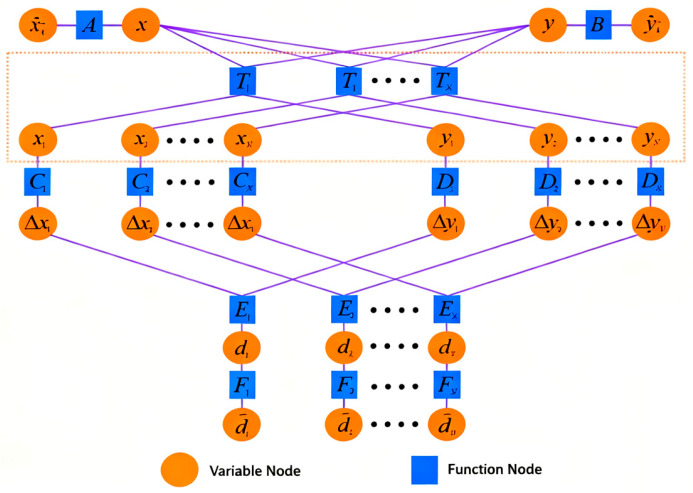
Factor graph of cooperative positioning system.

The factor graph model of the cooperative positioning algorithm contains *N* node groups, where *N* represents the number of observation data points received by the follower UUV, as shown in Figure 2. The ranging information d between the leader and follower UUVs is input into the factor graph through node E. The prior position estimates of the follower UUV are incorporated via nodes A and B. By applying the rotation matrix at node T in real time, the coordinates of both submarines participating in the factor graph computation are dynamically adjusted to maintain positional consistency, thereby reducing computational errors. Subsequently, nodes C and D convert the leader and follower UUV positions into x-y coordinate differences, which are then fused through node E to achieve an integrated spatial representation.

(1) Initialization

Determine the initial state at the beginning of cooperative positioning:

(12) $$\begin{array}{l} {\hat{x}}_{k}^{-}=x_{0},\sigma_{{\hat{x}}_{k}^{-}}^{2}=\sigma_{x_{0}}^{2}\\ {\hat{y}}_{k}^{-}=y_{0},\sigma_{{\hat{y}}_{k}^{-}}^{2}=\sigma_{y_{0}}^{2} \end{array}$$

(2) Time update for ${\hat{x}}_{k}^{-}$ and ${\hat{y}}_{k}^{-}$

For non-initial moments, the bearing can be calculated according to the following formula:

(13) $$\left\{\begin{array}{l} {\hat{x}}_{k}^{-}={\hat{x}}_{k-1}+{\hat{v}}_{k}\cos\theta_{k}\Delta t\\ {\hat{y}}_{k}^{-}={\hat{y}}_{k-1}+{\hat{v}}_{k}\sin\theta_{k}\Delta t \end{array}\right.$$

The expectation of velocity is $\mu_{v}$, and the variance is ${\sigma_{v}}^{2}$. The expectation of yaw is $\mu_{\theta }$, and the variance is ${\sigma_{\theta }}^{2}$. Since the velocity magnitude is independent of yaw, they are cov(v,θ)=0.

It can be calculated by the following formula:

(14) $$\begin{array}{l} E\left({\hat{x}}_{k}^{-}\right)=E\left({\hat{x}}_{k-1}\right)+\left(\mu_{v}\cos\mu_{\theta }-\frac{\sigma_{\theta }^{2}\mu_{v}\cos\mu_{\theta }}{2}\right)\Delta t\\ E\left({\hat{y}}_{k}^{-}\right)=E\left({\hat{y}}_{k-1}\right)+\left(\mu_{v}\sin\mu_{\theta }-\frac{\sigma_{\theta }^{2}\mu_{v}\sin\mu_{\theta }}{2}\right)\Delta t \end{array}$$

(15) $$\begin{array}{l} D\left({\hat{x}}_{k}^{-}\right)=D\left({\hat{x}}_{k-1}\right)+{\left(\cos\mu_{\theta }\Delta t\sigma_{v}\right)}^{2}+{\left(\mu_{v}\sin\mu_{\theta }\Delta t\sigma_{\theta }\right)}^{2}\\ D\left({\hat{y}}_{k}^{-}\right)=D\left({\hat{y}}_{k-1}\right)+{\left(\sin\mu_{\theta }\Delta t\sigma_{v}\right)}^{2}+{\left(\mu_{v}\cos\mu_{\theta }\Delta t\sigma_{\theta }\right)}^{2} \end{array}$$

The expectation and variance of variables ${\hat{x}}_{k}^{-}$ and ${\hat{y}}_{k}^{-}$ are updated by Equations (14) and (15).

(3) Measurement Update Based on Adaptive Weight Sum-Product

After introducing the transformation matrices of the function nodes $T_{n}$ (*n* = 1…*N*) for coordinate transformation, the variance of the estimated position undergoes corresponding changes. These changes are specifically reflected in the information transfer of the function node $T_{n}$. The probability density function transmitted from the function node $T_{n}$ to the variable node $x_{i}$ can be expressed as follows:

(16) $$N\left(x_{i},x\cos\theta +y\sin\theta ,\sigma_{x}^{2}\cos^{2}\theta +\sigma_{y}^{2}\sin^{2}\theta \right)$$

where $\sigma_{x}^{2}$ represents the variance passed from the variable node x to the function node $T_{n}$, and $\sigma_{y}^{2}$ indicates the variance passed from the variable node y to the function node $T_{n}$.

The probability density function passed from the function node $T_{n}$ to the variable node $y_{i}$ can be expressed as follows:

(17) $$N\left(y_{i},-x\sin\theta +y\cos\theta ,\sigma_{y}^{2}\cos^{2}\theta +\sigma_{x}^{2}\sin^{2}\theta \right)$$

among $\theta =\frac{\theta_{1}+\theta_{2}}{2},\theta_{1}=\arctan\left(\frac{y_{s}-y_{m}}{x_{s}-x_{m}}\right),\theta_{2}=\arctan\left(\frac{x_{m}-x_{s}}{y_{s}-y_{m}}\right)$.

According to Formula (13), it can be determined that the final estimated results of nodes x and *y* can be expressed as follows:

(18) $$\begin{array}{l} \mu (x)=\prod_{h\in N_{x}}\mu_{h\to x}\\ \mu (y)=\prod_{h\in N_{y}}\mu_{h\to y} \end{array}$$

Assuming that all variables are Gaussian distributions, after calculation and accumulation algorithm, the updated probability density functions of *x* and *y* are obtained as follows:

(19) $$\begin{array}{l} x\sim N\left(x,m_{x},\sigma_{x}^{2}\right)\\ y\sim N\left(y,m_{y},\sigma_{y}^{2}\right) \end{array}$$

where *m_x_*, $\sigma_{x}^{2}$, *m_y_*, and $\sigma_{y}^{2}$ can be obtained by the following formula:

(20) $$\begin{array}{l} \sigma_{x}^{2}=\frac{1}{\frac{1}{D\left({\hat{x}}_{k}^{-}\right)}+\sum_{n=1}^{N}\frac{\rho_{k}^{x_{n}}}{\sigma_{xn}^{2}}},\sigma_{y}^{2}=\frac{1}{\frac{1}{D\left({\hat{y}}_{k}^{-}\right)}+\sum_{n=1}^{N}\frac{\rho_{k}^{y_{n}}}{\sigma_{yn}^{2}}}\\ m_{x}=\sigma_{x}^{2}\left(\frac{{\hat{x}}_{k}^{-}}{D\left({\hat{x}}_{k}^{-}\right)}+\sum_{n=1}^{N}\frac{\rho_{k}^{x_{n}}m_{x_{n}}}{\sigma_{x_{n}}^{2}}\right),m_{y}=\sigma_{y}^{2}\left(\frac{{\hat{y}}_{k}^{-}}{D\left({\hat{y}}_{k}^{-}\right)}+\sum_{n=1}^{N}\frac{\rho_{k}^{y_{n}}m_{y_{n}}}{\sigma_{y_{n}}^{2}}\right) \end{array}$$

where *N* represents the number of distance information observed by the UUV at the same time; $m_{x_{n}}$ represents the expectation of the *x*-coordinate of the UUV position estimated from the observation information of the *n*-th leader UUV; $m_{y_{n}}$ represents the expectation of the *y*-coordinate of the UUV position estimated from the observation information of the *n*-th leader UUV; $\sigma_{x_{n}}^{2}$ represents the variance corresponding to $m_{x_{n}}$; $\sigma_{y_{n}}^{2}$ represents the variance corresponding to $m_{y_{n}}$; $\rho_{k}^{x_{n}}$ represents the information gain of the *x*-coordinate of the UUV position estimated by the *n*-th leader UUV observation information at time *k*; and $\rho_{k}^{y_{n}}$ represents the information gain of the *y*-coordinate of the UUV observed by the *n*-th leader UUV at time *k*.

Dolphins face a key “cocktail party problem” when coordinating navigation and predation: they must extract effective navigation information in real time from a complex sound field that includes peer echoes, environmental noise, and their own echoes. Biological studies have shown that dolphins respond to this challenge by dynamically adjusting the frequency, bandwidth, duration, and even directionality of their sonar pulses. For example, when perceiving increased interference, individuals tend to emit longer and more characteristic pulses to improve signal-to-noise ratio and may adjust the direction of the sound beam through head movements, essentially performing an “online recalibration” of their perception system based on environmental feedback.

In our FGAWSP algorithm, the continuous adaptive weight module based on residual evaluation is the mathematical modeling and engineering implementation of this biological intelligence. The algorithm considers each collaborative ranging observation as an “acoustic signal” to be evaluated. Traditional binary fault detection (accept or reject) is like dolphins can only choose to “fully listen” or “completely block” signals in a certain frequency band, which is obviously inefficient and not in line with biological reality. Our continuous weighting mechanism simulates the “gradient” judgment of signal credibility in dolphins: when the residual of an observation (analog signal anomaly) is small, it is given a weight close to 1 (“highly trusted and adopted”); as the residual increases, the weight smoothly decreases (“cautious reference, discounted adoption”); when the residual is extremely large, the weight approaches 0 (“judged as strong interference, basically ignored”). This process has a high degree of isomorphism with dolphins dynamically adjusting their perception strategies based on instantaneous acoustic environments through the core logic of “evaluation feedback regulation”.

We creatively propose a factor graph information fusion method based on adaptive weights and the product algorithm. When estimating the final position of the vessel, it constructs information gain adjustments $\rho_{k}^{x_{n}}$ and $\rho_{k}^{y_{n}}$ to improve fusion performance in the case of measurement anomalies. $\rho_{k}^{x_{n}}$ and $\rho_{k}^{y_{n}}$ remain a continuously changing function even after exceeding the threshold. When $\rho_{k}^{x_{n}}$ and $\rho_{k}^{y_{n}}$ are equal to 1, Equation (20) becomes the estimation result of the ordinary sum and product algorithm. Our designed $\rho_{k}^{x_{n}}$ and $\rho_{k}^{y_{n}}$ can be expressed as follows:

(21) $$\begin{array}{l} \rho_{k}^{x_{n}}=\left\{\begin{array}{c} 1\\ Y_{Dm}/\lambda_{k}^{x_{n}} \end{array}\right.\begin{array}{c} \lambda_{k}^{x_{n}}\leq Y_{Dm}\\ \lambda_{k}^{x_{n}}>Y_{Dm} \end{array}\\ \rho_{k}^{y_{n}}=\left\{\begin{array}{c} 1\\ Y_{Dm}/\lambda_{k}^{y_{n}} \end{array}\right.\begin{array}{c} \lambda_{k}^{y_{n}}\leq Y_{Dm}\\ \lambda_{k}^{y_{n}}>Y_{Dm} \end{array} \end{array}$$

where the initial value of $Y_{Dm}$ is selected based on empirical values and adaptively adjusted after ten measurement updates (using the median of the last ten times $\left(\lambda_{k}^{x_{n}}+\lambda_{k}^{y_{n}}\right)/2$), as expressed by the following equation:

(22) $$Y_{Dm}=\mathrm{med}(\begin{array}{cccc} \frac{\lambda_{k}^{x_{n}}+\lambda_{k}^{y_{n}}}{2} & \frac{\lambda_{k-1}^{x_{n}}+\lambda_{k-1}^{y_{n}}}{2} & \ldots & \frac{\lambda_{k-9}^{x_{n}}+\lambda_{k-9}^{y_{n}}}{2} \end{array})$$

where $\lambda_{k}^{x_{n}}$ and $\lambda_{k}^{y_{n}}$ can be expressed as follows:

(23) $$\lambda_{k}^{x_{n}}=\frac{{\left(m_{x_{n}}-{\hat{x}}_{k}^{-}\right)}^{2}}{D\left({\hat{x}}_{k}^{-}\right)+\sigma_{x_{n}}^{2}},\lambda_{k}^{y_{n}}=\frac{{\left(m_{y_{n}}-{\hat{y}}_{k}^{-}\right)}^{2}}{D\left({\hat{y}}_{k}^{-}\right)+\sigma_{y_{n}}^{2}}$$

In each update process, the estimation of the UUV position is carried out through Equation (23), and the expectation in Equation (23) is taken as the final position estimate of the UUV.

The information of other variable nodes and function nodes is updated according to the sum-product algorithm, which will not be introduced in this paper. To simplify the estimation process, the pseudo code of the FGAWSP algorithm can be expressed in Algorithm 1:
**Algorithm 1.** Pseudo code of the FGAWSP algorithm**Input: **$d_{i},X_{i}$ and
$Y_{i}$**Output**: pdf(*x*) and pdf(*y*)1. Initialization
$E\left({\hat{x}}_{k}^{-}\right),D\left({\hat{x}}_{k}^{-}\right),E\left({\hat{y}}_{k}^{-}\right),D\left({\hat{y}}_{k}^{-}\right)$2. **for**
*i* = 1 to *N*
**do**3.
$E\left(\Delta x_{i}^{-}\right)=X_{i}-{\hat{x}}_{k}^{-},D\left(\Delta x_{i}^{-}\right)=D\left({\hat{x}}_{k}^{-}\right)$4.
$E\left(\Delta y_{i}^{-}\right)=Y_{i}-{\hat{y}}_{k}^{-},D\left(\Delta y_{i}^{-}\right)=D\left({\hat{y}}_{k}^{-}\right)$5. compute pdf
$\left(\Delta y_{i}\right)$ using (10) in node
$E_{n}$6. compute pdf
$\left(\Delta x_{i}\right)$ using (10) in node
$E_{n}$7. compute pdf
$\left(x_{i}\right)$ using (16) in node
$C_{n}$8. compute pdf
$\left(y_{i}\right)$ using (17) in node
$D_{n}$9. **end for**10. compute pdf
(x) using (20) in node
x11. compute pdf
(y) using (20) in node
y12. **return** pdf(*x*) and pdf(*y*)

## 4. Simulations and Shipboard Experiment

### 4.1. Simulations

1. Simulation Condition Setting

**Figure 3:**
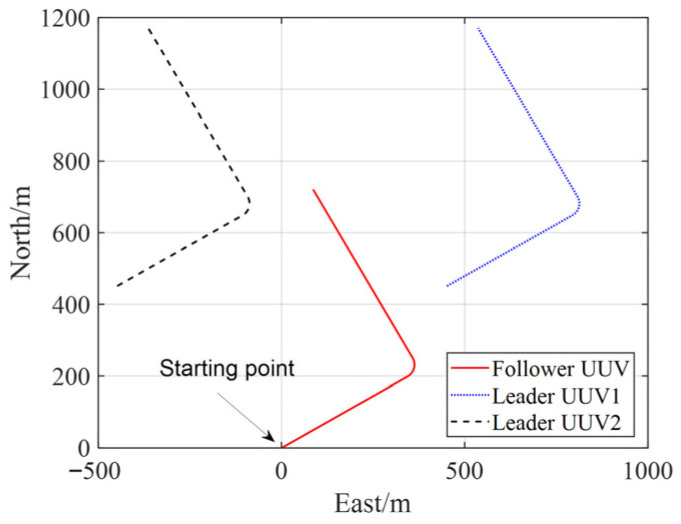
Trajectories of each UUV.

In the simulation, three UUVs are employed for cooperative positioning. Two UUVs as leader UUVs (Leader UUV1 with coordinates (450, 450) and Leader UUV2 with (−450, 450)), while the follower UUV starts at (0, 0). All UUVs maintain a navigation speed of 2 m per second throughout the 500 s simulation period, with their trajectories illustrated in Figure 3.

**Figure 4:**
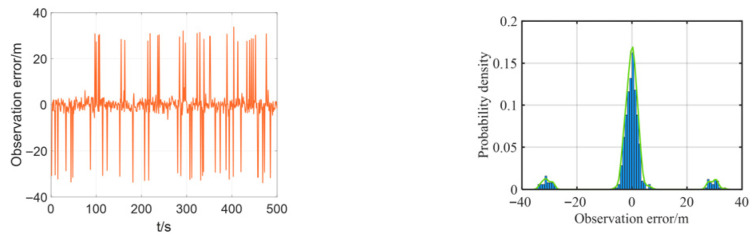
Observation error of the follower UUV and its probability density curve.

**Figure 5:**
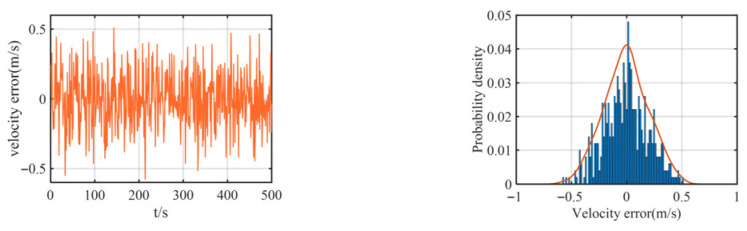
Speed error of the follower UUV and its probability density curve.

**Figure 6:**
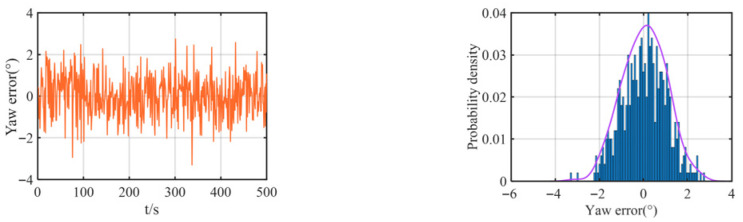
Yaw error of the follower UUV and its probability density curve.

The multipath effect is a fundamental physical phenomenon in underwater acoustics, where a transmitted signal travels to the receiver via multiple paths of different lengths due to reflections from boundaries like the sea surface and bottom, as well as refraction and scattering within the water column. This is not merely an attenuation issue. The core problem arises because the signals arriving via these indirect, longer paths are delayed copies of the direct path signal. When they superimpose at the receiver, they cause constructive or destructive interference, leading to severe signal fading. For ranging systems, this poses a critical challenge: if the receiver inadvertently locks onto a strong reflected path rather than the direct path, the measured signal time-of-flight is artificially inflated, resulting in a large, positive bias in the calculated distance—a classic outlier. This effect is particularly pronounced in shallow waters, where boundary reflections are intense. Consequently, the statistics of the resulting ranging errors deviate significantly from a Gaussian distribution, exhibiting a characteristic “heavy-tailed” property where large errors occur with non-negligible probability. Therefore, we added some outliers in the simulation to simulate heavy-tailed noise. In the simulation, we added some noise as velocity, yaw, and observation (relative distance) errors. The observation errors and their probability distributions between the two leader boats and the follower boat are shown in Figure 4, the velocity error and its probability distribution are shown in Figure 5, and the yaw error and its distribution are shown in Figure 6.

As shown in Figure 5 and Figure 6, the added yaw and velocity noise are Gaussian distributions: the velocity error follows a Gaussian distribution with a mean of 0 m/s and a standard deviation of 0.2 m/s, while the yaw error follows a Gaussian distribution with a mean of 0 m/s and a standard deviation of 1°. The ranging errors of both the lead vessel and the trailing vessel are additionally simulated with a 30 m deviation based on a Gaussian distribution with a mean of 0 m/s and a standard deviation of 2 m/s, representing the practical heavy-tailed noise characteristics encountered in actual navigation scenarios.

2. Simulation Results

**Figure 7:**
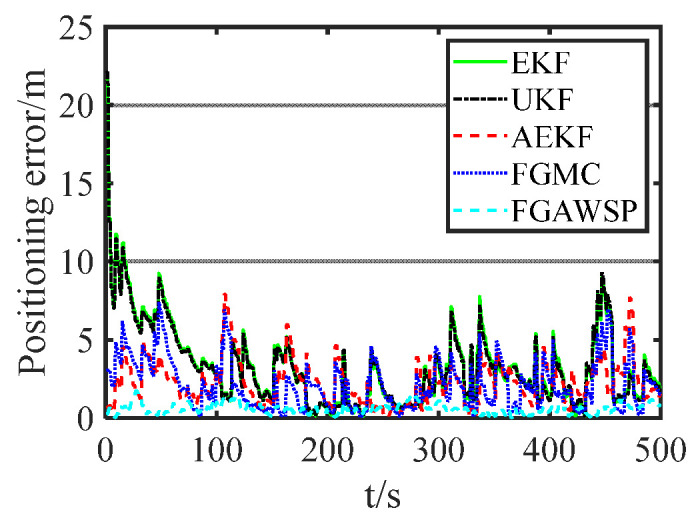
Positioning error curves of different algorithms.

**Figure 8:**
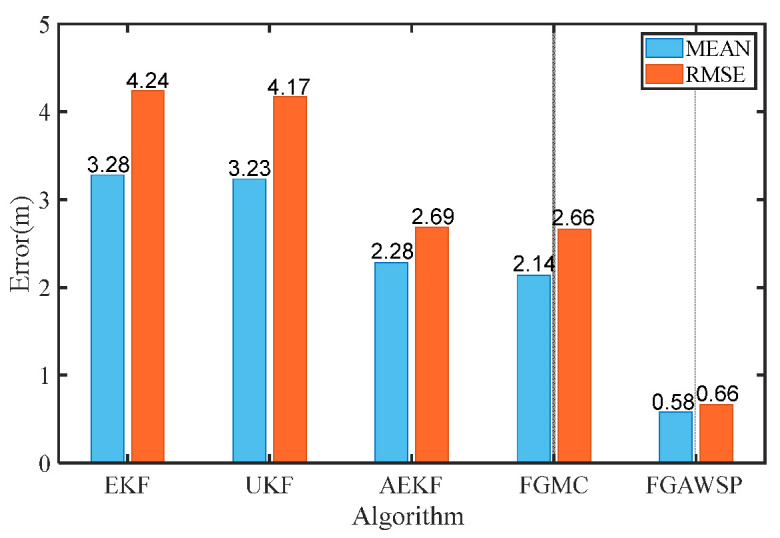
Comparison of positioning errors of different algorithms.

In the simulation, the proposed algorithm was compared with traditional methods. Figure 7 displays the positioning error curves of different algorithms, showing that the FGAWSP algorithm exhibits smaller positioning errors compared to other methods. To visually compare their performance, we calculated the mean and root mean square errors (RMSEs) of each algorithm, as illustrated in Figure 8.

We selected EKF, UKF, AEKF, and FGMC as comparison algorithms in the simulations and experiments mainly based on the following considerations. EKF and UKF are classic benchmark algorithms for solving nonlinear filtering problems and are widely used in UUV collaborative positioning research. Using them as comparison baselines can help readers intuitively understand the degree of improvement in algorithm performance. AEKF (Adaptive EKF) represents an improved filtering method for system uncertainty, which enhances robustness by adaptively adjusting noise statistical characteristics. FGMC (Factor Graph Algorithm Based on Maximum Correlation Entropy) is an advanced factor graph method proposed in recent years specifically for handling non-Gaussian, heavy-tailed noise, which is highly relevant to the core problem studied in this paper. These algorithms collectively cover different technical routes from traditional nonlinear filtering and adaptive filtering to modern robust graph optimization, and have good representativeness.

In order to compare the computational complexity of different algorithms, we calculated the time required for the same computer to run different algorithms under the same simulation conditions, as shown in Table 1. From the table, it can be seen that the algorithm proposed in this article has a lower computational complexity than EKF, UKF, and AEKF algorithms, and is slightly larger than the FGMC algorithm. The algorithm takes less computation time than EKF and can be applied in real-time systems.

The proposed FGAWSP algorithm demonstrates a mean positioning error of 0.58 m and a root mean square error (RMSE) of 0.66 m, significantly outperforming other algorithms. Compared with the EKF, UKFAEKF, and FGMC algorithms, the RMSE of FGAWSP achieves reductions of 84.43%, 84.17%, 75.46%, and 75.19%, respectively. The simulation experiments validate the effectiveness of the proposed algorithm.

To avoid any randomness in the simulation results, we conducted an additional eight simulations, and the results are shown in Table 2. We can see from Table 2 that the algorithm still outperforms other algorithms after multiple simulations and comparisons.

### 4.2. Shipboard Experiment

1. Shipboard Experiment Condition

To further validate the performance of the proposed FGAWSP algorithm in practical cooperative positioning scenarios, we conducted offline verification using historical shipborne experimental data. The shipboard experiment was carried out in the waters of the Taihu Lake, Wuxi, in August. The average depth of this water area is about 2.6 m, which is a typical shallow water experimental environment. The on-site water temperature was about 28 °C, and the salinity of the water was relatively low, belonging to a freshwater environment. During the experiment, the lake surface was relatively calm, with an estimated surface flow velocity of less than 0.2 m per second. This shallow water environment characteristic significantly exacerbates multipath effects and boundary reflections during the propagation of underwater acoustic signals, becoming the main environmental factor causing observation noise to exhibit a significant non-Gaussian, heavy-tailed distribution.

**Figure 9:**
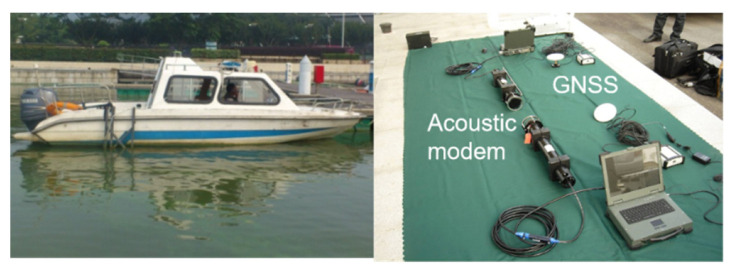
Experimental ship and main experimental sensors.

During the trials, GNSS was employed as the positioning reference for the unmanned underwater vehicle (UUV). Doppler velocimeters measured the UUV velocity, while magnetic compasses determined its azimuth. The distance between the two leader UUVs and the follower UUV was measured using underwater acoustic ranging equipment, with position and distance information transmitted to the follower boat via underwater communication systems. The sampling frequency of the satellite positioning system in the shipborne test was 1 Hz, and the communication frequency of the underwater communication equipment was 0.2 Hz. Due to the distance between UUVs being about 100 m, the communication delay was less than 0.1 s, which has little impact on navigation at low speeds. The experimental equipment is shown in Figure 9.

The sensor indicators used in the test are shown in Table 3. The positioning accuracy of the RTK, used as the reference GNSS, is 15 mm, the azimuth accuracy of the magnetic compass is 2°, the speed measurement accuracy of the Doppler meter is ±0.3%, and the working range of the acoustic modem is 8000 m.

2. Experimental Results

**Figure 10:**
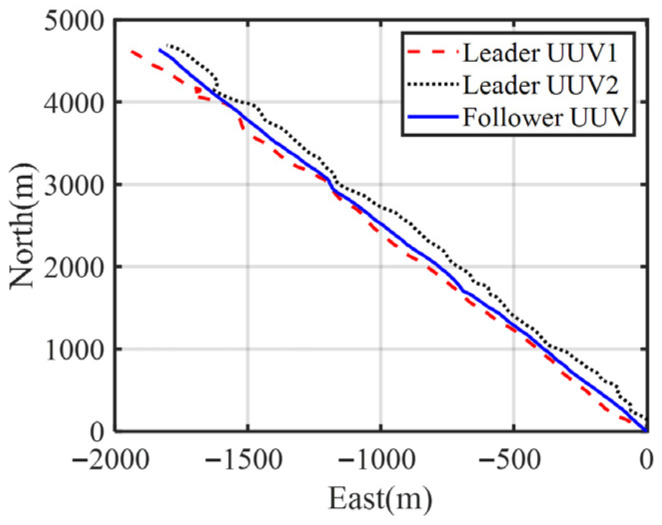
Trajectories of each UUV in the experiment.

**Figure 11:**
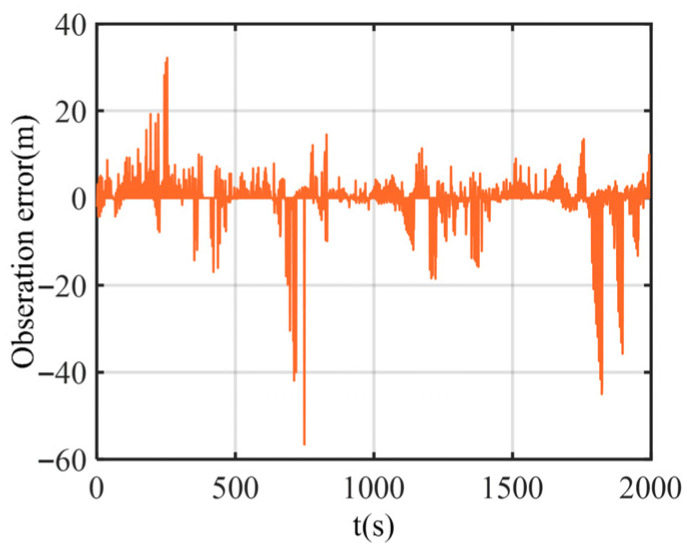
Observation error of the follower UUV.

The trajectories of each boat in the test are shown in Figure 10, and the test time was 2000 s. Figure 11 shows the noise measured by the acoustic ranging equipment between the two leader boats and the follower boat. As can be seen from the figure, there are some large outliers in the ranging errors.

**Figure 12:**
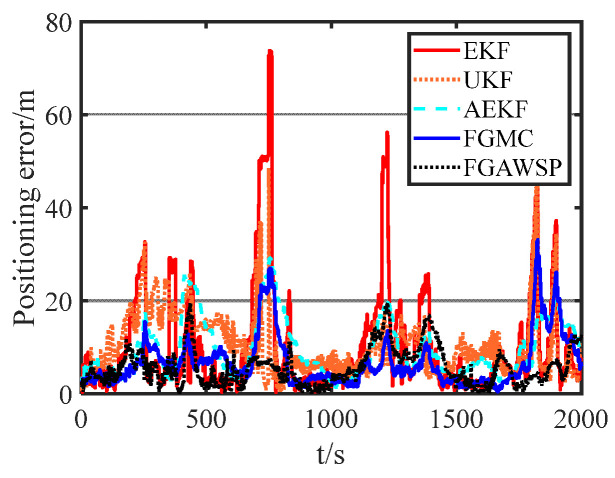
Different algorithm positioning error curves of follower UUV.

**Figure 13:**
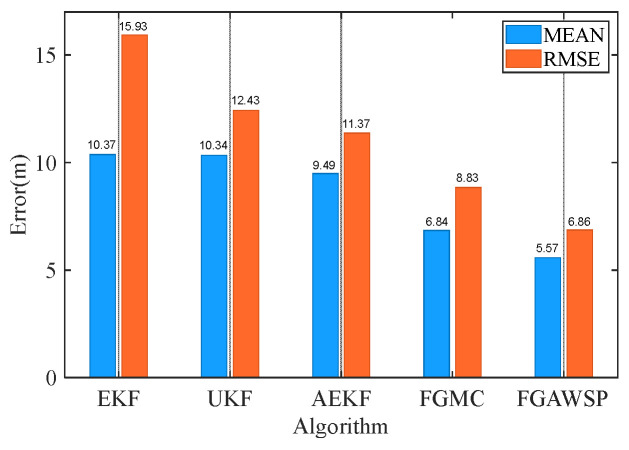
Statistical results of positioning errors for different algorithms of follower UUV.

Figure 12 compares the performance of different algorithms when processing experimental data. The figure clearly shows that the traditional EKF algorithm is most affected by ranging errors. The FGMC method, which uses the maximum correlation entropy criterion as its cost function, effectively reduces the impact of outliers. In contrast, the proposed FGAWSP algorithm demonstrates excellent performance when handling large outliers. To further visually compare the results of different algorithms, Figure 13 presents statistics on the mean and root mean square error (RMSE) of positioning errors across various methods.

The analysis results indicate that, in the shipborne experiment, the performance differences between FGAWSP and EKF, UKF, AEKF, and FGMC reached a statistically significant level. The *t*-test *p*-values for different algorithms with the FGAWSP method are shown in Table 4.

The root mean square error (RMSE) of the proposed FGAWSP algorithm in this study is 6.86 m, while the EKF, UKF, AEKF, and FGMC algorithms show RMSE values of 15.93 m, 12.43 m, 11.37 m, and 8.83 m, respectively. Compared with these methods, our algorithm achieves significant improvements, with reductions of 56.94%, 44.81%, 39.67%, and 22.31% in RMSE. Experimental results demonstrate that FGAWSP exhibits exceptional robustness when processing real-world experimental data, outperforming traditional collaborative localization algorithms by a clear margin.

## 5. Conclusions

In this paper, we develop a factor graph-based adaptive cooperative positioning algorithm for heavy-tailed noise. We first establish a factor graph model to address UUV cooperative positioning challenges, then innovatively design a novel factor graph estimation mechanism combining adaptive weights and product algorithms. By leveraging residual information to dynamically adjust fusion weights in the factor graph, the proposed method effectively mitigates the impact of outliers in heavy-tailed noise while enhancing the robustness of cooperative positioning. Extensive validation through simulation experiments and shipboard tests demonstrates the effectiveness of our FGAWSP algorithm. Simulation results show a 75.19% error reduction compared to the FGMC algorithm, with shipboard testing achieving a 22.31% improvement. These outcomes collectively validate the practical value of our algorithm in real-world engineering applications.

This study recognizes that the currently proposed FGAWSP algorithm has certain limitations. Firstly, the algorithm model is based on the assumption that lateral velocity can be ignored, which may introduce errors in strong lateral flow or high maneuverability scenarios. Secondly, the research focuses on two-dimensional horizontal positioning, and expansion to three-dimensional space will increase the computational burden and bring new observability issues. In addition, performance verification of the algorithm is mainly carried out in shallow lake environments, and its applicability and robustness in complex open sea areas need further verification. In response to these shortcomings, future research will focus on enhancing models to adapt to highly dynamic environments, expanding the algorithm to three-dimensional space, exploring deep learning hybrid architectures integrated with traditional filtering, and conducting extensive validation under conditions closer to real ocean operations, in order to comprehensively promote the practical development of this technology.

## Figures and Tables

**Table 1:** Running time of each algorithm.

Algorithm	EKF	UKF	AEKF	FGMC	FGAWSP
Running time	0.0345 s	0.0832 s	0.0634 s	0.0268 s	0.0336 s

**Table 2:** Positioning error of each algorithm.

Algorithm	EKF	UKF	AEKF	FGMC	FGAWSP
RMSE	4.35 m	4.15 m	2.83 m	2.73 m	0.74 m
	4.61 m	4.26 m	2.91 m	2.88 m	0.71 m
	4.29 m	4.06 m	2.81 m	2.75 m	0.65 m
	4.88 m	4.61 m	3.01 m	2.93 m	0.86 m
	4.75 m	4.28 m	2.96 m	2.67 m	0.82 m
	4.25 m	4.19 m	2.65 m	2.61 m	0.62 m
	4.11 m	4.06 m	2.48 m	2.36 m	0.59 m
	4.92 m	4.32 m	2.99 m	2.97 m	0.93 m

**Table 3:** Types and performance parameters of sensors utilized.

Sensor	Performance
GNSS	Single-point positioning accuracy: 1.5 m RTK positioning accuracy: 15 mm
Compass	yaw accuracy: 2°
DVL	velocity accuracy: ±0.3%
Acoustic Modem	operation range: 8000 m

**Table 4:** The *t*-test *p*-values for different algorithms with the FGAWSP method.

Algorithm	EKF	UKF	AEKF	FGMC
T-test *p*-values of different algorithms with the FGAWSP method	1.4894 × 10 ^−65^	1.2171 × 10 ^−154^	1.0505 × 10 ^−124^	1.9978 × 10^−20^

## Data Availability

The raw data supporting the conclusions of this article will be made available by the authors on request.
